# The need for a balanced hospital-based care (HBC) and home- and community-based care (HCBC) approach for mitigating COVID-19 pandemic in sub-Saharan Africa

**DOI:** 10.11604/pamj.2021.38.196.25559

**Published:** 2021-02-22

**Authors:** Harris Onywera, Lamech Malagho Mwapagha, Nei-Yuan Hsiao

**Affiliations:** 1 Institute of Infectious Disease and Molecular Medicine, University of Cape Town, Cape Town, South Africa,; 2 Division of Medical Virology, Department of Pathology, Faculty of Health Sciences, University of Cape Town, Cape Town, South Africa,; 3 Department of Natural and Applied Sciences, Faculty of Health and Applied Sciences, Namibia University of Science and Technology, Windhoek, Namibia,; 4 Department of Clinical Virology, National Health Laboratory Service (NHLS), Groote Schuur Hospital, Cape Town, South Africa

**Keywords:** SARS-CoV-2, COVID-19, pandemic, healthcare, HBC, HCBC, sub-Saharan Africa

## Abstract

The onslaught of COVID-19 pandemic has greatly overwhelmed some of the best healthcare systems in the world. Medical practitioners working in hospitals at the epicenters of COVID-19 pandemic have emphasized on the need to manage mildly ill and convalescent COVID-19 patients at home or community facilities rather than at hospitals during a pandemic. In this article, we highlight that a standardized home- and community-based (HCBC) approach for management of COVID-19 patients will be a key component for preparing hospitals in sub-Saharan Africa (SSA) for a potential surge in COVID-19 cases. So far, based on the trajectory of infection, we think that SSA seems to have a window of opportunity, albeit narrowing, for implementing HCBC. However, there are challenges that will need to be addressed in order to implement and maintain HCBC. Successful implementation and maintenance of HCBC in SSA will require international agencies and key donors to work closely with the national governments; providing them with policy, technical, and financial assistance. Home- and community-based care (HCBC) is also important because it can play a role in advocacy, education, training, and health promotion during COVID-19 pandemic. We further underscore the need for a delicate balance between HCBC and hospital-based care (HBC) approach as well as with COVID-19 mitigation and suppression measures in order to reduce the risk of SARS-CoV-2 community transmission and allow optimal continuity of the HBC. We conclude by emphasizing once again that, for countries in SSA to adequately prepare for the worst-case scenario of COVID-19 pandemic in the absence of a cure, policy makers of member states need to act collectively and fast.

## Perspectives

As COVID-19 pandemic sweeps across the globe, it continues to precipitate inconceivable sociocultural, health, and economic mayhems. By mid-April 2020, over 2.0 million people were infected with SARS-CoV-2 globally; with the US and Western Europe rapidly overtaking China by approximately eight- to twelve-fold to become the new epicenters of the pandemic. This upward infection trajectory, especially in countries such as Italy and Spain might not be surprising as it is believed that at the onset of the SARS-CoV-2 outbreak, countries´ mitigation efforts to control the epidemic were “too little” and “too late” [1]. As of 14 August 17H00 (15: 00 GMT), over 21.1 million people were infected with SARS-CoV-2 globally; with the number of confirmed cases in several countries, including Russia, India, Brazil and South Africa to mention a few, greatly surpassing the number of cases in China [2]. Although the importation risk of COVID-19 to Africa was estimated to be eleven times lower than that to Europe [3], the pandemic has already found a foothold in Africa, especially in South Africa. Notwithstanding, this global outbreak displays an uneven geographical distribution in Africa. So far, the continent has 1,089,894 confirmed cases and 24,752 deaths [2]. Each of these represents a small yet increasing proportion of the global confirmed cases and deaths (5.2% and 3.3% respectively). There has been growing concerns about the impact of COVID-19 pandemic on the African continent. For example, based on modelling and population-based intervention analyses, Walker and colleagues estimated that Africa could have 1.0 billion SARS-CoV-2 infections and 2.5 million deaths this year, following an unmitigated pandemic [4]. However, the three-month infection trajectory of COVID-19 cases in African countries seems to be quite unique compared to the epidemic in other parts of the world. The infections are not growing faster as predicted. We think that these slow infection trajectories in Africa could be due to: i) timely implementation of containment measures (in order to reduce COVID-19 importation risk), ii) low SARS-CoV-2/COVID-19 testing capacity (thus, underestimating the cases), iii) warm weather (which could be reducing or precluding the spread of infection), iv) Africa´s young population (which could offer protection against the infection), and v) pre-existing BCG vaccination programmes (which is thought could reduce the impact of the pandemic). In spite of the unique infection trajectories, African countries should still be concerned over the rising COVID-19 cases. This is because the healthcare systems in these countries are weak despite the need for healthcare prioritization by their governments. Concurrent with this, there is currently no specific therapy for COVID-19 despite the ongoing global advances in the development of COVID-19 drugs and vaccines.

The onslaught of the pandemic has greatly strained some of the best healthcare systems in the world. For instance, in Daegu and Gyeongbuk (South Korea), the rapid surge in COVID-19 cases resulted in shortage of hospital beds. Consequently, this forced some of the COVID-19 patients to stay and/or die at their homes while awaiting admissions [5]. In Lombardy (Italy), one of the initial responses to the outbreak was to increase the intensive care unit (ICU) surge capacity [6]. However, the hospital-based care (HBC), including ICU care, rapidly reached its saturation and was at the verge of collapsing [7]. The surge in COVID-19 cases resulted in overcrowding in hospitals, fatigued healthcare workers (HCWs), in-hospital SARS-CoV-2 transmission, prioritization of ICU care according to patients´ chances of surviving, struggle to deliver regular healthcare services, suspension of vaccination programmes, and lack or inadequate vital medical supplies and equipment [7]. An unmanageable COVID-19 pandemic, compounded by inadequate personal protective equipment (PPE), may potentially expose HCWs to COVID-19. The strain posed on HBC systems by the raid surge in COVID-19 patients therefore begged the question of whether out-of-hospital care approaches, specifically home- and community-based care (HCBC), could be exploited to provide adequate healthcare to COVID-19 patients without overloading the HBC system. To achieve this, several medical practitioners working at COVID-19 epicenters in Italy emphasized that, “in a pandemic, patient-centered care is inadequate and must be replaced by community-centered care” [7]. Parallel with this appeal, the World Health organization (WHO) published its interim guidance for provision of safe home care for COVID-19 patients “when inpatient care is unavailable and unsafe” [8]. Fairly recently, South Korea implemented a resource-saving and cost-effective out-of-hospital cohort care termed as community treatment center (CTC). CTCs are designed for treating mild cases of COVID-19 while allowing for cohort isolation (so as to prevent transmission) and restricting hospitalization to patients with severe symptoms [5]. With the continued spread of SARS-CoV-2 infection in sub-Saharan Africa (SSA) and signs for possible relaxation of lockdowns and curfews lifting in order to save the economy, particularly in the absence of specific antiviral treatment for COVID-19 and limited HBC capacity, there is an urgent need for careful consideration of HCBC for management of COVID-19 patients.

**Feasibility and relevance of home- and community-based care and patient management in sub-Saharan Africa (requirements and challenges):** but how feasible and pertinent is HCBC in SSA, especially amid minimal supplies of medical resources (including PPE) and inadequate COVID-19 isolation facilities? As already known, there is lack of hospital capacity in SSA [4,9] concomitant with increased burden of nosocomial infections as well as communicable and non-communicable diseases [9], e.g., malaria, HIV/AIDS, tuberculosis, diabetes, chronic respiratory conditions, and cancer. Some of these diseases are associated with poor clinical outcomes of COVID-19 [10]. Nosocomial infections have been reported among hospitalized COVID-19 patients [10], and may cause financial burden (due to extended hospitalizations) and death of patients. Given the unprecedented healthcare challenges staring at the fragile healthcare systems in SSA, it therefore seems logical to assume that HBC in SSA will be inadequate for a surged COVID-19 pandemic. Even if surged COVID-19 cases do not occur in countries in SSA, the few available hospitals could be overwhelmed by the COVID-19 cases. Thus, it would be advisable for the major stakeholders, including policy makers, in the healthcare system in the respective countries in SSA to tackle COVID-19 pandemic from a HCBC approach. This could be intertwined with community screening and testing. Home- and community-based care (HCBC) will be a key component of preparing healthcare facilities for a potential surge in COVID-19 cases, in addition to eliminating or minimizing further burden on the already stretched HBC systems.

The success of HCBC will greatly depend on the availability of basic shelter, mobile clinics/field hospitals, community facilities (e.g., repurposed hotels, gymnasiums, and stadiums), essential medical resources (medicines, beds, and equipment), reliable road infrastructure, good infection, prevention and control (IPC) measures, proper healthcare risk waste mismanagement as well as good national policy, political will, and governance. Additionally, HCBC will need a new cadre of community staff with the spirit of humanity and COVID-19 patients attended to irrespective of their income and medical covers. Despite the need for rapid widespread campaign and implementation of HCBC in countries in SSA during COVID-19 pandemic, there are certain healthcare issues and challenges [9,11,12] that could potentially impede its adoption. Adoption of HCBC may be limited by: i) working poverty and lack of basic sanitation facilities and supporting infrastructures, ii) little, delayed, and slow efforts by the stakeholders in healthcare in adopting HCB, iii) shortages and unequal distribution of trained staff, iv) frequent periodic supply shortages and stock-outs of vital medicines and limited medical equipment, v) high burden of disease epidemics, vi) unwillingness of COVID-19 patients and their contacts to be enrolled in HCBC due to stigma against COVID-19, vii) looming and cascading concerns about the possibility of adverse health outcomes during the COVID-19 management period, viii) safety of home- and community-based carers, which may limit the amount of work performed by community HCWs, ix) conflict between modern medicine and alternative medicine, including traditional medicine, x) difficulty in establishing a balancing HCBC and HBC as well as COVID-19 countermeasures, xi) environmental hazards due to poor hygiene and IPC measures, and healthcare risk mismanagement, xii) poor-record keeping which might compromise patient care and its continuum, xiii) different COVID-19 patient needs, and increased patient needs and expectation, xiv) lack of education and training for COVID-19 patients and community HCWs, xv) possibility of medical litigation due to patient negligence, xvi) ailing economy and limited funding parallel with management and leadership crisis.

Therefore, the success of HCBC will greatly depend on the availability of basic shelter, mobile clinics/field hospitals, community facilities (e.g., repurposed hotels, gymnasiums, and stadiums), essential medical resources (medicines, beds, and equipment), reliable road infrastructure, good IPC measures, safe healthcare risk waste mismanagement as well as good national policy, political will, and governance. Additionally, HCBC will need a new cadre of empowered community staff with the spirit of humanity and COVID-19 patients attended to irrespective of their income and medical covers. Instituted national programmes will be needed to monitor and evaluate the impact of HCBC. There are lessons that could be adopted from pre-existing home-based care policies for fighting disease epidemics such as HIV. To implement and sustain an effective HCBC for COVID-19 pandemic in SSA, international agencies (e.g., African Union Commission, Africa Centers for Disease Control and Prevention (Africa CDC) and WHO) and key donors might need to work closely with the national governments, providing them with policy and technical guidance, and financial support. Under the HCBC approach, standardized procedures could be used to triage COVID-19 patients to apposite healthcare needs based on the patterns of disease progression. This could be bolstered by use of point-of-care testing, such as lactate dehydrogenase/glucose ratio, C-reactive protein, and lymphocyte count, as well as pulse oximetry in order to determine patients who need hospitalization. Severity of lymphocytopenia for example, has been posited to show severity of COVID-19 [10]. Persons with suspected COVID-19 and those with mild symptoms and no risk factors (underlying chronic conditions) who do not need hospitalization can be managed under HCBC in accordance with international health guidelines [8], until their symptoms resolve and SARS-CoV-2 tests are negative.

**What role can home- and community-based care play in the fight against SARS-CoV-2/COVID-19?** Home- and community-based care can play a role in advocacy, education, training, and health promotion. Such include regularly mobilizing the community and taking part in campaigns geared towards educating the community about the potential risk of COVID-19 and empowering them on the importance of mitigation and suppression measures - hand hygiene practices, respiratory etiquette, social distancing, lockdowns, etc. COVID-19 information material and resources (including digital advocacy) should be readily available in local languages in order to capture a targeted and wider audience. These, together with patient education on COVID-19, will further mitigate SARS-CoV-2 transmission. Education and training should be supplemented by good counselling that prevents panic, stigmatization, social discrimination, and patient-attack associated with COVID-19.

**Need for a balanced hospital-based care and home- and community-based care approach:** with the advent of HCBC and consideration to adopt it by countries such as South Africa and Kenya, there is need to establish a balance between HCBC and HBC approach for COVID-19 in SSA. Home- and community-based care (HCBC) requires a well-coordinated and robust communication link with HBC. Furthermore, a balance of HCBC approach with community isolation and IPC measures for COVID-19 ought to be considered. This is primarily intended to reduce the risk of SARS-CoV-2 onward transmission and “flatten the curve”, hence allowing optimal continuity of the HBC. In tandem with COVID-19 containment and mitigation policies, HCBC could also encompass drone technology and telemedicine, in order to deliver healthcare services to COVID-19 patients (recent cases, convalescents, and survivors), while preventing HCWs from potential COVID-19 and nosocomial infections. Patients under HCBC could be clinically managed with available therapies that do not necessarily require hospitalization and new therapies as good clinical data becomes available. More importantly, comorbidities should be taken into considerations during HCBC. Regular follow-ups will be required to ensure that COVID-19 patients fully recover or are timely transferred to hospitals if necessary. Home- and community-based care will need community HCWs and specific household members to be trained on how to manage COVID-19 patients and themselves at home and community facilities according to the current WHO recommendations [8].

## Conclusion

Because the pandemic has not yet stamped its authority in Africa, SSA seems to have a critical window for implementing HCBC, though this is closing with time as the number of COVID-19 cases continues to rise. Nonetheless, it should once again be emphasized that in order for Africa to prepare for the worst-case scenario of COVID-19 pandemic in the absence of a cure, policy makers of member states need to “act collectively, and fast” [13]. To counter the pandemic, HCBC could be used concomitantly with HBC and population-wide COVID-19 mitigation and suppression interventions such as wider social distancing. While HCBC represents a feasible and pertinent approach for preventing the HBC from becoming inadequate for surged COVID-19 pandemic in SSA, it is not a single magic bullet for mitigating the pandemic.
